# Digital transformation of health and care to sustain Planetary Health: The MASK proof-of-concept for airway diseases—POLLAR symposium under the auspices of Finland’s Presidency of the EU, 2019 and MACVIA-France, Global Alliance against Chronic Respiratory Diseases (GARD, WH0) demonstration project, Reference Site Collaborative Network of the European Innovation Partnership on Active and Healthy Ageing

**DOI:** 10.1186/s13601-020-00321-2

**Published:** 2020-06-19

**Authors:** Jean Bousquet, Josep M. Anto, Tari Haahtela, Pekka Jousilahti, Marina Erhola, Xavier Basagaña, Wienczyslawa Czarlewski, Mikaëla Odemyr, Susanna Palkonen, Mikael Sofiev, César Velasco, Anna Bedbrook, Rodrigo Delgado, Rostislav Kouznetsov, Mika Mäkelä, Yuliia Palamarchuk, Kimmo Saarinen, Erja Tommila, Erkka Valovirta, Tuula Vasankari, Torsten Zuberbier, Isabella Annesi-Maesano, Samuel Benveniste, Eve Mathieu-Dupas, Jean-Louis Pépin, Robert Picard, Stéphane Zeng, Julia Ayache, Nuria Calves Venturos, Yann Micheli, Ingrid Jullian-Desayes, Daniel Laune

**Affiliations:** 1 Charité, Universitätsmedizin Berlin, Humboldt-Universität zu Berlin, Berlin, Germany; 2 Department of Dermatology and Allergy, Berlin Institute of Health, Comprehensive Allergy Center, Berlin, Germany; 3 MACVIA-France, Montpellier, France; 4 ISGlobAL, Centre for Research in Environmental Epidemiology (CREAL), Barcelona, Spain; 5 Universitat Pompeu Fabra (UPF), Barcelona, Spain; 6 CIBER Epidemiología y Salud Pública (CIBERESP), Barcelona, Spain; 7 Skin and Allergy Hospital, Helsinki University Hospital and Helsinki University, Helsinki, Finland; 8 Finnish Institute for Health and Welfare, Helsinki, Finland; 9 Medical Consulting Czarlewski, Levallois, and MASK-air, Montpellier, France; 10 EFA European Federation of Allergy and Airways Diseases Patients’ Associations, Brussels, Belgium; 11 Finnish Meteorological Institute (FMI), Helsinki, Finland; 12 Agency for Health Quality and Assessment of Catalonia (AQuAS), Barcelona, Spain; 13 Obukhov Institute for Atmospheric Physics, Moscow, Russia; 14 Université Grenoble Alpes, Grenoble, France; 15 Allergy, Skin and Asthma Federation, Helsinki, Finland; 16 Finnish Lung Health Association (Filha), Helsinki, Finland; 17 Department of Lung Diseases and Clinical Immunology, University of Turku and Terveystalo Allergy Clinic, Turku, Finland; 18 FILHA, Finnish Lung Health Association, Helsinki, and Turku University, Turku, Finland; 19 Epidemiology of Allergic and Respiratory Diseases, Department Institute Pierre Louis of Epidemiology and Public Health, INSERM and Sorbonne Universités, Medical School Saint Antoine, Paris, France; 20 National Center of Expertise in Cognitive Stimulation (CEN STIMCO), Broca Hospital, Paris, France; 21 Mines ParisTech CRI-PSL Research University, Fontainebleau, France; 22 KYomed INNOV, Montpellier, France; 23 Laboratoire HP2, Grenoble, INSERM, U1042 and CHU de Grenoble, Université Grenoble Alpes, Grenoble, France; 24 Conseil Général de l’Economie Ministère de l’Economie, de l’Industrie et du Numérique, Paris, France; 25 Bull DSAS, Echirolles, France; 26 Institute of Psychology, Memory and Cognition Laboratory, Paris Descartes University, Sorbonne Paris Cité, Boulogne Billancourt, France; 27 CHU Arnaud de Villeneuve, 371 Avenue du Doyen Gaston Giraud, 34295 Montpellier Cedex 5, France

**Keywords:** Asthma, Planetary health, mHealth, Finnish Allergy Programme, ARIA

## Abstract

In December 2019, a conference entitled “Europe That Protects: Safeguarding Our Planet, Safeguarding Our Health” was held in Helsinki. It was co-organized by the Finnish Institute for Health and Welfare, the Finnish Environment Institute and the European Commission, under the auspices of Finland’s Presidency of the EU. As a side event, a symposium organized as the final POLLAR (Impact of air POLLution on Asthma and Rhinitis) meeting explored the digital transformation of health and care to sustain planetary health in airway diseases. The Finnish Allergy Programme collaborates with MASK (Mobile Airways Sentinel NetworK) and can be considered as a proof-of-concept to impact Planetary Health. The Good Practice of DG Santé (The Directorate-General for Health and Food Safety) on digitally-enabled, patient-centred care pathways is in line with the objectives of the Finnish Allergy Programme. The ARIACARE-Digital network has been deployed in 25 countries. It represents an example of the digital cross-border exchange of real-world data and experience with the aim to improve patient care. The integration of information technology tools for climate, weather, air pollution and aerobiology in mobile Health applications will enable the development of an alert system. Citizens will thus be informed about personal environmental threats, which may also be linked to indicators of Planetary Health and sustainability. The digital transformation of the public health policy was also proposed, following the experience of the Agency for Health Quality and Assessment of Catalonia (AQuAS).

## Introduction

Most economies are struggling to deliver cost-effective, high-quality healthcare. There is an urgent need for the healthcare system to leverage developments in digital health in order to improve cost-effectiveness [1]. The term “digital health” includes advanced medical technologies, disruptive innovations and digital communication tools aiming to provide best practices [2, 3].

A conference entitled “Europe That Protects: Safeguarding Our Planet, Safeguarding Our Health” was based on the new concept of the interactions between human health and the health of the planet. It brought stakeholders together, including researchers, policy makers and regulators. The aim was to identify and discuss (i) the scientific challenges for harvesting and enhancing the benefits of a sound environment for human health, and (ii) the challenges to overcome environmental threats to human health [4]. The main outcome consisted of recommendations on research and innovation priorities for the EU and Member States to enable collaborative work to protect both human health and our planet. This event was co-organized by the Finnish Institute for Health and Welfare, the Finnish Environment Institute and the European Commission, under the auspices of Finland’s Presidency of the EU in 2019.

The aim of POLLAR (Impact of Air POLLution on Asthma and Rhinitis, EIT Health) [5, 6] is to combine emerging technologies in order to understand the effect of air pollution on allergic rhinitis and its impact on sleep, work and asthma. The objective is to propose novel care pathways at the EU level integrating online pollution data and symptoms of allergy in the already existing sentinel network.

During this conference, a symposium organized as the final POLLAR meeting was held to explore the digital transformation of health and care—using airway diseases as an example—and how it relates to planetary health. Implementation of the messages and goals of the Finnish Allergy Programme 2008–2018 was shown as a model, which could be further enhanced by the digital transformation of health.

## Planetary Health

In 2015, a new approach called “Planetary Health” was proposed by a Rockefeller Foundation-Lancet Commission. Planetary Health was defined as the achievement of the highest attainable standard of health, wellbeing and equity worldwide through judicious attention to the human systems—political, economic and social—that shape Earth’s natural system limits within which humanity can flourish [7, 8]. Since its inception, the Planetary Health approach has received substantial attention with an increasing presence in the academic community.

However, how to deploy the Planetary Health approach in the health care system remains a challenge. First, the health care system needs to join the societal efforts to drastically cut CO_2_ emissions in order to reach the goal of the Paris agreement. The Intergovernmental Panel on Climate Change (IPCC) 1.5º report in 2018 claimed that current emission needs to be reduced by 40% in 2030 and should be reduced to zero by 2050. There is a large potential for health care systems contributing to these goals. The adoption of Planetary Health in health care and public health services can be facilitated by identifying the strategies that result in clear interactions between human health and the health of the Planet.

Some examples can be proposed for respiratory diseases. Smoking, which is one of the leading causes of preventable mortality worldwide with 7 million deaths annually, has been shown to be a relevant cause of environmental degradation due to deforestation, intensive use of pesticides and other poor environmental practices. Green-house emissions, caused by cultivating tobacco, correspond to the full emissions of some single countries [9, 10]. Health care and technology can be considered good for health (assuming appropriate use) but it has a significant negative impact on the planet. In Australia, it accounts for about 7% of all CO_2_ emissions, mainly attributable to hospitals and medicines [11]. Regarding respiratory and allergic diseases, the Environmental Audit Committee of the UK parliament has requested that by 2022 at least 50% of prescribed inhalers should be of low global warming potential (GWP) [12]. Planting trees in a city is good for human and planetary health but planting allergenic trees is not good for human health. These and other examples show how health care and public health can adopt a Planetary Health transformative strategy and contribute to stopping global warming and the loss of natural resources in which human health can sustainably progress.

However, how to bring the potential of the planetary health strategies to the digital transformation of health care is a challenge. Currently, there are multiple apps for capturing either dietary or travel behaviour and estimating the associated carbon cost. However, most of these apps focus on one or a few individual issues like travel or diet and are of mixed quality [13].

The POLLAR effort to bring both air pollution and pollen season data to a digital application, along with information of symptoms and medication followed by allergy patients, is a new innovation. The data can be further supplemented by climate models and forecasts. The integration of data from different sources and a new kind of modelling for personal use can improve the health of patients and spread information needed for action to sustain planetary health. The idea, with disease specific information and data collecting, might be scalable for many other non-communicable diseases.

## Deployment of the Finnish Allergy Programme, a proof-of-concept to impact Planetary Health

The Finnish Allergy Programme (2008–2018) revisited the allergy and asthma paradigm by turning avoidance strategy into tolerance/resilience strategy and improving nature relatedness [14, 15].

Environmental and lifestyle factors are responsible for most of the so-called allergy and asthma epidemic since the second world war. That is why *immune tolerance* and *allergy health* were promoted through a *Nature Step* in resetting the connection between humans and the natural environment [16]. Promoting *physical activity*, reducing *air pollution* and stopping *smoking* were also central. Both primary and secondary (tertiary) prevention were in focus.

The 10-year programme defined goals, tools and measures as well as follow-up of outcome. A long-term educational campaign was implemented both for healthcare professionals and the lay public. The burden of allergy and asthma in Finland started to decline with less medicalisation, fewer allergy diets, less severe asthma, and fewer costs [17]. The Finnish experience can be deployed in *European Union* countries having efficient and accessible public healthcare. The good educational level of citizens allows new information to penetrate effectively. The key words are: motivate and organize. Do not develop anything, be precise: say what you are going to do, by what means, and how you are going to follow the outcome. The allergy and asthma burden can be reduced, and very likely the burden of many other non-communicable diseases which are on the rise everywhere in urban societies. Promoting public health also goes together with the safeguarding of our planet [7]. The Finnish Allergy Programme is sustaining planetary health by many aspects (Table 1).

**Table 1:** Potential of the Finnish Allergy Programme to address Planetary Health

Finnish Allergy Programme	Impact on Planetary Health
Endorse health, not allergy	Endorse the Planet
Improve contact with nature	Contribute to conserving and regenerating nature
Strengthen tolerance/resilience	Understand and strengthen the role of biodiversity
Avoid allergens only if mandatory	Promote Planet sustainable diets
Prevent allergies	Reduce the planetary impact of medical treatments and harmful chemicals in planet to human, human to planet chain
Recognize and treat severe allergies early	Reduce the planetary impact of avoidable health care wastage
Improve air quality	Reduce air pollution, both outdoors and indoors
Stop smoking	Eradicate smoking and its environmental impacts

## The MASK digital transformation of health and care for airway diseases

Over the past 20 years, ARIA (Allergic Rhinitis and its Impact on Asthma) [18] has evolved, with strong political commitment, from the first multi-morbidity guideline in respiratory diseases to a proof-of-concept of the digital transformation of health and care for the management of patients with long-term conditions.

As a proof-of-concept for chronic disease care, MASK, the Phase 3 ARIA project [19, 20], in collaboration with professional and patient organizations in the field of allergy and airway diseases, is proposing real-life integrated care pathways (ICPs) centred around the patient with rhinitis and asthma multi-morbidity. These next-generation ICPs represent a prism through which the potential for digitally transforming healthcare can be viewed. MASK is a Good Practice of DG Santé on digitally-enabled, patient-centred ICPs [21].

Although environmental factors play a major role in allergic diseases, they have never been included in guidelines or recommendations. Based on the results of POLLAR [5], ARIA next-generation ICPs are embedding exposure to environmental factors such as pollen and air pollution. Furthermore, novel approaches such as artificial intelligence are being used. As there is increasing evidence that patients’ choices and behaviours have an impact on the Planet, this will be the background of ARIA Planetary Health [7].

## Next-generation care pathways for allergic rhinitis

ICPs are structured multi-disciplinary care plans detailing the key steps of patient care [22]. They promote the translation of guideline recommendations into local protocols and their application to clinical practice. ICPs have been proposed with a focus on mHealth technologies that should enhance self-management and adherence to guidelines and ICPs. They represent an important topic of the digital transformation of health and care.

**Fig. 1:**
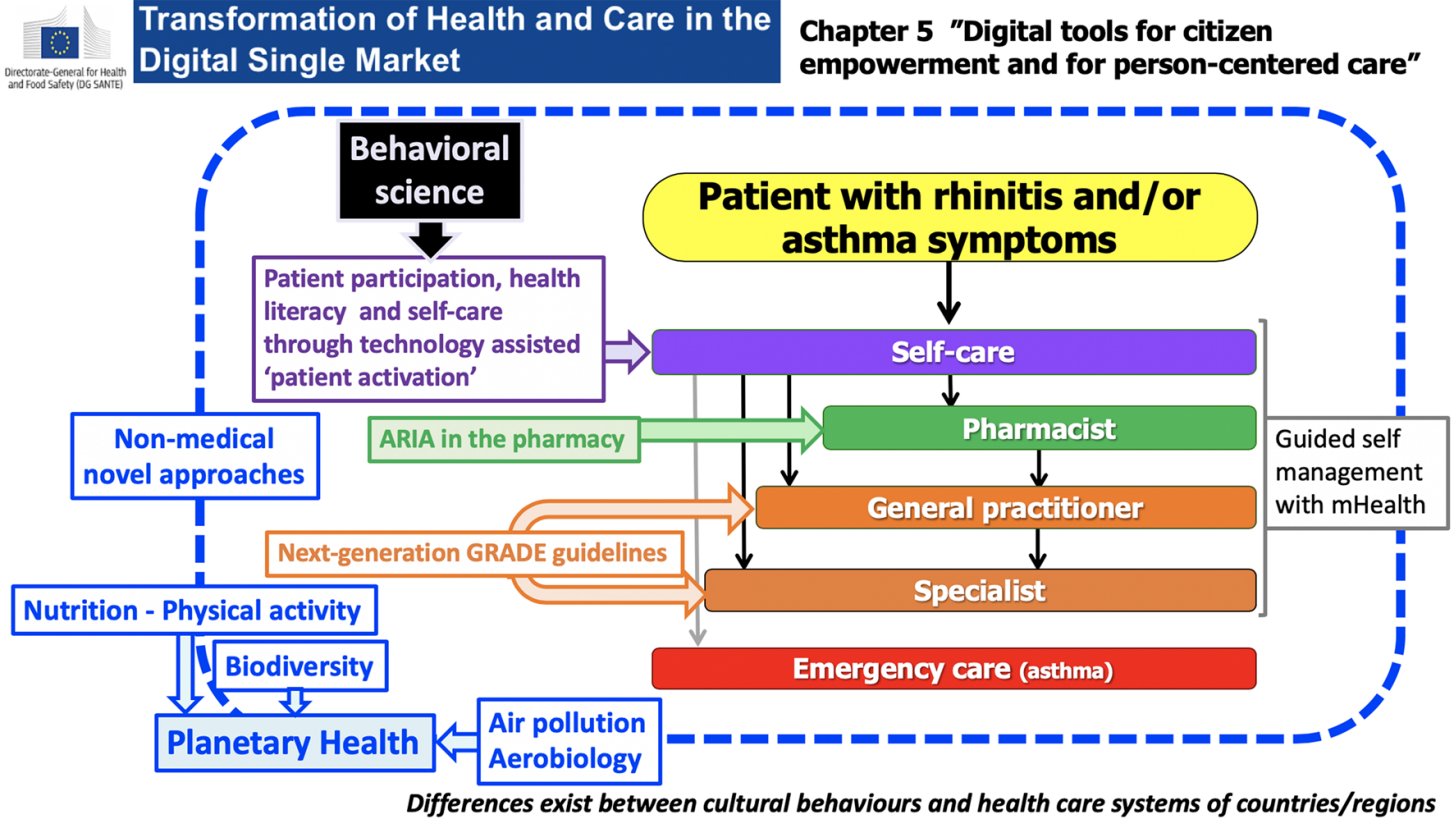
Next-generation care pathways integrating Planetary Health sustainability (from [24])

Next-generation care pathways for allergic rhinitis represent an innovative digitally-enabled, patient-centred approach for ICPs [23]. They have been proposed by ARIA Phase 4 for rhinitis and asthma multi-morbidity to be scaled up to chronic diseases. Five aspects of ICPs have particularly been developed (Fig. 1).

1. Self-care: Patient participation, health literacy and self-care through technology-assisted patient activation [25].
2. Pharmacist: Most patients with allergic rhinitis use OTC medications and a guide for the pharmacist, already proposed by ARIA in 2004 [26], has been updated [27]. A pocket guide is pending.
3. ICPs for allergen immunotherapy [28]: Only guidelines for AIT were previously proposed. This is the first ICP. The ARIA-EAACI pocket guide is available.
4. The next-generation guidelines for allergic rhinitis have assessed GRADE recommendations in AR and asthma using real-world evidence (RWE) [23]. RWE includes not only randomized-controlled trials on treatment effects, but also real-world data (RWD) obtained by mHealth tools—including MASK—and chamber studies [29] in order to confirm effectiveness or refine current recommendations [30]. MASK results were used to refine the GRADE guidelines [31]. This appears to be the first attempt to test GRADE recommendations with RWE.
5. It is very important to include non-medical interventions in ICPs, such as feeding cats with egg produced anti-Fel d1antibodies to reduce their allergenicity [32].

The Impact on Planetary Health represents the sixth approach. Several aspects of Planetary Health are included in next-generation ICPs—such as biodiversity, exercise, air pollution and aerobiology—but also actions by municipalities to improve human and Planetary Health (e.g. the ECARF action plan in Berlin). The POLLAR predictive model of pollen seasons and air pollution index are one of the examples.

These next-generation ICPs have been used to develop value added medicine.

The Finnish Allergy Programme goals are in line with many results of the MASK (Mobile Airways Sentinel NetworK) digital platform on allergies and asthma [19] (Table 2).

**Table 2:** Set-up goals for health care of the Finnish Allergy Programme and digital health

Goals	Indicators	Digital transformation of health: MASK	
Prevent allergy	Asthma, rhinitis and atopic eczema prevalence reduced 20%		
Improve tolerance	Food allergy diets in day-care and schools reduced 40%	Cat food containing anti-Fel d1 may help to keep cats at the homes of allergic patients	[32]
Improve allergy diagnostics	Skin testing (prick and patch tests) in certified testing centres	A screening test for asthma, COPD and rhinitis helps in the diagnosis of allergies and in sending the patients to certified allergy centres	[33], in preparation
Reduce work-related allergies	Occupational allergies (asthma, rhinitis, contact dermatitis) reduced 40%	Daily VAS work assessment	[34]
Focus on severe allergies and treat in time	Good allergy practice defined, asthma emergency visits reduced 40%	MASK: Good Practice of DG Santé Adherence to treatment* Efficacy of medications* Next-generation care pathways Next-generation guidelines* Value added medicine* (*: proof-of-concept in rhinitis)	[21] [31, 35] [31, 36] [24, 28] [24, 28]
Reduce allergy and asthma costs	Allergy-related total costs (direct and indirect) reduced 10%	EQ-5D, WPAI-AS	[37]

## ARIACARE centres of excellence

In ARIA Phase 4 (change management for airway diseases) [21, 38], a network of centres of excellence (CoE) is being organized. GA^2^LEN ARIACARE is part of the GA^2^LEN CoE that already includes UCARE (for urticaria) and ADCARE (for atopic dermatitis) [39]. Accreditation follows the UCARE proposals, and ARIA members will be able to apply. Other groups will also be able to join ARIACARE.

ARIACARE-Digital is a novel network with the aim to implement the digital transformation of health and care in airway diseases. Both members of MASK and others can join this network. ARIACARE-Digital is being developed with EAACI and has links with GA^2^LEN but is independent. Three levels are proposed for these centres (regular, gold and platinum) with a review each year. Numerous benefits are expected from this innovation.

Digital health is progressing towards more interoperability for the digital cross border exchange of health data in Europe. The ARIACARE-Digital network is already deployed in 25 countries around the world using the same standard operating procedures (SOPs) for allergic diseases and asthma and represents a proof-of-concept for digital cross-border exchange.

## Citizens’ views on the respiratory targets of planetary health

Patients with respiratory disease are the sentinels of air quality since poor air quality indoors and outdoors directly impacts their disease and can cause or exacerbate respiratory allergy, asthma or COPD. Therefore, together with pulmonary and public health doctors, they have been the first advocates for the right to clean air and better air quality standards. People with asthma and COPD in Europe state that their top priority is for governments to do more regarding air pollution [40]. Recently, the whole society, in particular young people, have become aware and risen up to combat climate change. According to WHO, air pollution needs to be at the core of the climate change action [41]. However, people’s health is not sufficiently put at the core of planetary health as being inherently interlinked. Health is among the top priorities for European citizens at national level and, for both EU and national level, concerns regarding climate change and environment are on the rise [42]. The “Green Deal” is one of the top priorities for the new European Commission [43]. The Finnish Council Presidency priority for Economy of Wellbeing integrates “health in all policies” [44].

Technological developments enable personal monitoring for air pollution—including pollen—combined with disease management. Citizens have become active actors for the environment, as well as for their personal health.

Planetary and human health go together, and citizens, no longer only patients, are at the core of this change. The digital transformation of healthcare to improve adherence and patient and citizen participation is here. However, the healthcare systems are falling behind, as citizens are leading with the use of apps.

There is an opportunity for interlinking individual and respiratory health to planetary health.

## Integration of IT tools with citizen science to promote Planetary Health

Citizen science represents a way of engaging citizens in science and of empowering them to understand, prioritize and tackle society challenges by co-creating scientific results that lead to new knowledge and action [45]. Citizens’ interests, creativity, diversity and experience can contribute with new ideas and can help to promote new research areas or existing ones that are closer to citizens’ interests. Still, many citizen science projects basically count on citizens just as data providers. An increase in projects with the highest involvement of citizens, including orienting research questions, has the potential to contribute to Planetary Health. Actually, many citizen science projects have dealt with environmental issues, especially environmental monitoring, showing an underlying interest of citizens in protecting the planet [46].

Likewise, citizens are concerned by their health, as shown by the increase of health monitoring with apps and sensors. The availability of new cheap sensors and apps for environmental monitoring and for measuring health parameters, like the Allergy Diary—MASK-air App [19], facilitates the creation of new citizen science studies linking environment and health. However, such studies face extra challenges compared to other citizen science projects [47]. Some of these challenges will be discussed during the presentation. These include the need for ethical approvals and informed consent forms; potential biases due to non-representativeness or self-selection; that public health issues are usually topics of public dispute; how to ensure that citizens have adequate competencies and capabilities to conduct the work and that the data collected and the inference made with it have sufficient quality. In addition, is important to manage citizens’ expectations, as they may expect solutions to a problem (not always possible), the study may produce unexpected or null results, and anyway the evidence from a single study is definitive and should be weighed with other available evidence.

## Integration of IT tools for climate, weather, air pollution and aerobiology

There are several models and integration technologies that bring together the key drivers of human pollen-related allergy and asthma. They include meteorological models, atmospheric composition and air quality models, pollen prediction models, pollen real-time monitoring techniques, and the mobile applications interacting with the allergy sufferers, collecting their self-assessment reports and delivering forecasts of their personalized allergy risk.

The POLLAR system is constructed around the MASK-air mobile application and its allergy symptom database. It is integrated with the Symptom Forecasting Model developed within the PASYFO (Personalized Allergy Symptoms Forecasting System) project of Copernicus Atmospheric Monitoring Service, which also supplies the meteorological, air quality and pollen information for Europe. Additional pollen and global air quality forecasts are generated by the SILAM (System for integrated modelling of atmospheric composition) model of the Finnish Meteorological Institute (FMI) [48–50]. Another novel component is real-time pollen monitoring, which is emerging in several European countries and being established also in Finland within the PS4A project of the Academy of Finland.

The key outcome of the system—the allergy risk forecasts based on the patients’ self-assessment and forecasts of environmental parameters—was established a few years ago as the basis of the forecasting setup of the system. It has recently been developed for POLLAR and embedded in the MASK app.

## Digital transformation in the health public policy

The Agency for Health Quality and Assessment of Catalonia (AQuAS) aims to carry out data evaluation and analysis to generate relevant and reliable information, promoting health and enhancing the sustainability of the Catalan health system. AQuAS has supported the Ministry of Health decision-making for better performance in safety, efficacy and efficiency, e.g. of public procurement. This has been done mainly by innovative health data analysis, health technology assessment and citizens’ or professionals’ consultations.

The Agency leads a strategical project on data analysis, the Public Data Analysis Program for Health Research and Innovation (PADRIS, http://aquas.gencat.cat/en/ambits/analitica-dades/padris/). A multidisciplinary team provides community health data of 7.5 million citizens, adding value through data to research, innovation and health assessment. According to the legal and regulatory framework, ethical principles and transparency, the programme provides access to anonymized data for around 50 public projects every year. Real World Data is generated by all health providers from the Catalan health service and stored on the Integrated Catalan health data system, known as SISCAT (*Sistema sanitari integral d’utilització pública de Catalunya*, http://sac.gencat.cat/sacgencat/AppJava/organigrama.jsp?codi=2803&jq=200040).

PADRIS is also an initiative positioning Catalonia in the international information society arena, moving towards a new specialized care model to promote research and transfer the results to clinical practice.

Findings of observational studies, RWE and registry data all require judicious evaluation when used to assess treatment effects. Health care decisions improve if this data reservoir provides insights beyond those addressed by randomized controlled trials. Due to the need of clinical decision support tools, the use of RWE has been essential. Catalonia is currently using RWD for evidence based decision making in different scenarios from the management to the clinical level. One remarkable case is the predictive model for the monitoring of epidemic seasons in Catalonia. Predictive mathematic models have been used for the potential early detection of epidemic patterns. Some examples are: the real-time predictive seasonal influenza model, developed by the Public Health Agency of Catalonia, and the asthma predictive seasonal model, which is currently being explored by AQuAS.

The real-time spatial predictive model of the ILI (Influenza Like Illness) incidence rate in Catalonia provides 1- and 2-week forecasts. These include historical and real-time data from different sources: the statutory reporting disease system, the flu sentinel surveillance system, the official meteorology service, microbiology diagnostics data and Google Flu Trends. The model is a useful decision-making tool although evaluation is difficult due to uncertainty. The accuracy of prediction at 1 and 2 weeks was above 80% globally, but was lower during the peak epidemic period [51].

Asthma is a common chronic respiratory condition in Spain, with a prevalence of 5% in adults and 10% in children. The vast majority of cases are diagnosed during childhood and 60–75% of asthma cases have an allergic origin.

In order to tackle the asthma epidemic, AQuAS is exploring a new predictive model oriented to health systems decision making. The aim is to design an algorithm to predict asthma peaks within time and space and to quantify the temporal patterns for acute symptoms. The model will integrate data from SISCAT clinical health records from 2008 to 2018, historical and real-time data from the electronical drug prescription registry as well as meteorological data. This kind of RWD predictive tool returns value to the Catalan society. It offers the opportunity to design preventive measures, plan healthcare needs, improve patient outcomes, and guide new research solutions.

## Data Availability

Not applicable.

## Abbreviations

AQuAS: Agency for Health Quality and Assessment of Catalonia
ARIA: Allergic Rhinitis and its Impact on Asthma
CoE: Centre of excellence
COPD: Chronic obstructive pulmonary disease
EAACI: European Academy of Allergy and Clinical Immunology
ECARF: European Centre for Allergy Research Foundation
EQ-5D: EuroQol
EU: European Union
GA2LEN: Global Allergy and Asthma European Network
ICP: Integrated care pathway
MACVIA: Contre les MAladies Chroniques pour un VIeillissement Actif
MASK: Mobile Airways Sentinel networK
POLLAR: Impact of air POLLution on Asthma and Rhinitis
OTC: Over-the-counter
PASYFO: Personalized allergy symptoms forecasting system
RWD: Real-world data
RWE: Real-world evidence
SILAM: System for integrated modelling of atmospheric composition
VAS: Visual analogue scale
WPAI-AS: Work productivity and activity
